# Low Temperature Extends the Lifespan of *Bursaphelenchus xylophilus* through the cGMP Pathway

**DOI:** 10.3390/ijms18112320

**Published:** 2017-11-03

**Authors:** Bowen Wang, Ling Ma, Feng Wang, Buyong Wang, Xin Hao, Jiayao Xu, Yan Ma

**Affiliations:** 1 College of Forestry, Northeast Forestry University, Harbin 150040, China; wangbowen6@126.com (B.W.); wangbuyong89@163.com (B.W.); xinhao@nefu.edu.cn (X.H); guooguoo@126.com (J.X.); 2 College of Management, Harbin University of Commerce, Harbin 150028, China

**Keywords:** *Bursaphelenchus xylophilus*, pine wilt disease, cGMP pathway, low temperature, lifespan extension

## Abstract

The causal agent of pine wilt disease, pine wood nematode (PWN) (*Bursaphelenchus xylophilus*), revealed extended lifespan at low temperature. To discover the molecular mechanism of this phenomenon, we attempted to study the molecular characterization, transcript abundance, and functions of three genes of the cyclic guanosine monophosphate (cGMP) pathway from *B. xylophilus*. Three cGMP pathway genes were identified from *B. xylophilus*. Bioinformatic software was utilized to analyze the characteristics of the three putative proteins. Function of the three genes in cold tolerance was studied with RNA interference (RNAi). The results showed that the deduced protein of Bx-DAF-11 has an adenylate and guanylate cyclase catalytic domain, indicating an ability to bind to extracellular ligands and synthesizing cGMP. Both Bx-TAX-2 and Bx-TAX-4 have cyclic nucleotide-binding domains and ion transport protein domains, illustrating that they are cGMP-gated ion channels. The transcript level of *Bx-daf-11*, *Bx-tax-2*, and *Bx-tax-4* increased at low temperature. The survival rates of three gene silenced *B. xylophilus* revealed a significant decrease at low temperature. This study illustrated that the cGMP pathway plays a key role in low-temperature-induced lifespan extension in *B. xylophilus*.

## 1. Introduction

Native species can live in their natural niche, yet invasive spices are more aggressive. Invasive species have a wide range of adaptation to various environmental factors [1]. Among those factors, temperature is the most crucial one for the formation of a stable population in the potential suitable area [2]. Low-temperature-induced diapause is an important condition for invasive species’ adaptation to a new ecological environment. The diapause states of invasive organisms are usually accompanied by life extension and morphological and secondary metabolic changes, which improve the survival rate in extreme environmental conditions [3,4,5].

As one of the most devastating invasive species in the world, *B. xylophilus* is the causal agent of pine wilt disease, which causes enormous ecological and financial losses by damaging the pine forest resources in Asia, North America, and Europe [6,7,8]. It has been reported that pine wood nematode (PWN) has infested the area where the climate is relatively cold, and the infestation area will continue expanding to colder regions of Asia and Europe [9,10,11]. Researchers have studied the morphology and secondary metabolism changes of low-temperature-induced lifespan extension in *B. xylophilus* before [12]. However, the molecular mechanisms of low-temperature-induced lifespan extension in *B. xylophilus* have not been studied.

*Caenorhabditis elegans* (*C. elegans*) is an excellent model organism in neurobiology research. The mechanism of temperature sensing has been widely studied in this organism [13,14,15,16,17]. Notably, some highly conserved cold-sensing related pathways including the TRPA-1 pathway [13], the estrogen signaling pathway [18], the insulin-signaling pathway [17], the cGMP pathway [16], and so on have been widely studied in *C. elegans*, which gives considerable guidance in the research of *B. xylophilus*. We hypothesize that there are similar temperature-detecting molecular mechanisms between *C. elegans* and *B. xylophilus*. It has been reported that the cGMP pathway is involved in regulating the process of low temperature signal transduction in *C. elegans* [16]. Three genes from the cGMP pathway in *C. elegans*, *daf-11*, *tax-2*, and *tax-4*, encode transmembrane guanylyl cyclase, cyclic nucleotides-gated channels β subunits, and cyclic nucleotides-gated channels α subunits, respectively [15,19,20,21]. Mutants of *daf-11*, *tax-4*, and *tax-2* showed increments of survival rates in low temperature [17]. Based on the *B. xylophilus* genome [22], we identified three *B. xylophilus* orthologous genes named *Bx-daf-11*, *Bx-tax-2*, and *Bx-tax-4*, which may have similar functions with *daf-11*, *tax-2*, and *tax-4* in defending against low temperature. This article is aimed to further research the function and expression patterns of *Bx-daf-11*, *Bx-tax-2*, and *Bx-tax-4* from *B. xylophilus* in response to low temperature.

## 2. Results

### 2.1. Cloning of the Three cGMP Genes and Alignment of Deduced Amino Acids

**Figure 1:**
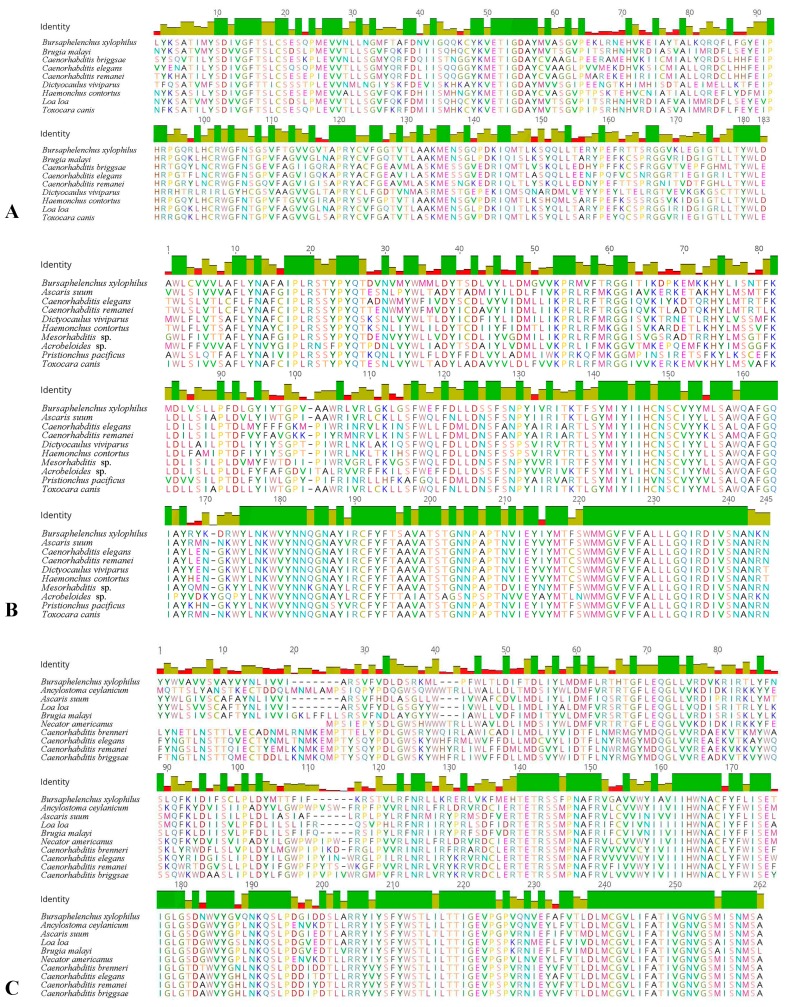
Alignment of *B. xylophilus* Bx-DAF-11, Bx-TAX-2, and Bx-TAX-4 with homologs identified from NCBI. (**A**) Comparison for protein homology of Bx-DAF-11 from *Brugia malayi* (CRZ24751.1), *Caenorhabditis briggsae* (XP_002636586.1), *C. elegans* (NP_505960.3), *C. remanei* (XP_003114238.1), *Dictyocaulus viviparus* (KJH45027.1), *Haemonchus contortus* (CDJ86375.1), *Loa loa* (XP_020304885.1), and *Toxocara canis* (KHN73536.1); (**B**) Comparison for protein homology of Bx-TAX-2 from *Ascaris suum* (ERG83679.1), *C. elegans* (AAB41492.1), *C. remanei* (ACQ44009.1), *D. viviparus* (KJH52997.1), *H. contortus* (CDJ81705.1), *Mesorhabditis* sp. (API61682.1), and *Acrobeloides* sp. (API61683.1); (**C**) Comparison for protein homology of Bx-TAX-4 from *Ancylostoma ceylanicum* (EYC38988.1), *A. suum* (ERG86438.1), *L. loa* (XP_003138541.1), *B. malayi* (CDP95269.1), *Necator americanus* (XP_013302261.1), *C. brenneri* (EGT33630.1), *C. elegans* (NP_499033.1), *C. remanei* (XP_003112989.1), and *C. briggsae* (XP_002642523.1). Note: Red, yellow, and green indicate variant amino acid residues (identity < 50%), highly conserved amino acid residues (50% ≤ identity < 100%), and invariant amino acid residues (identity = 100%), respectively.

**Figure 2:**
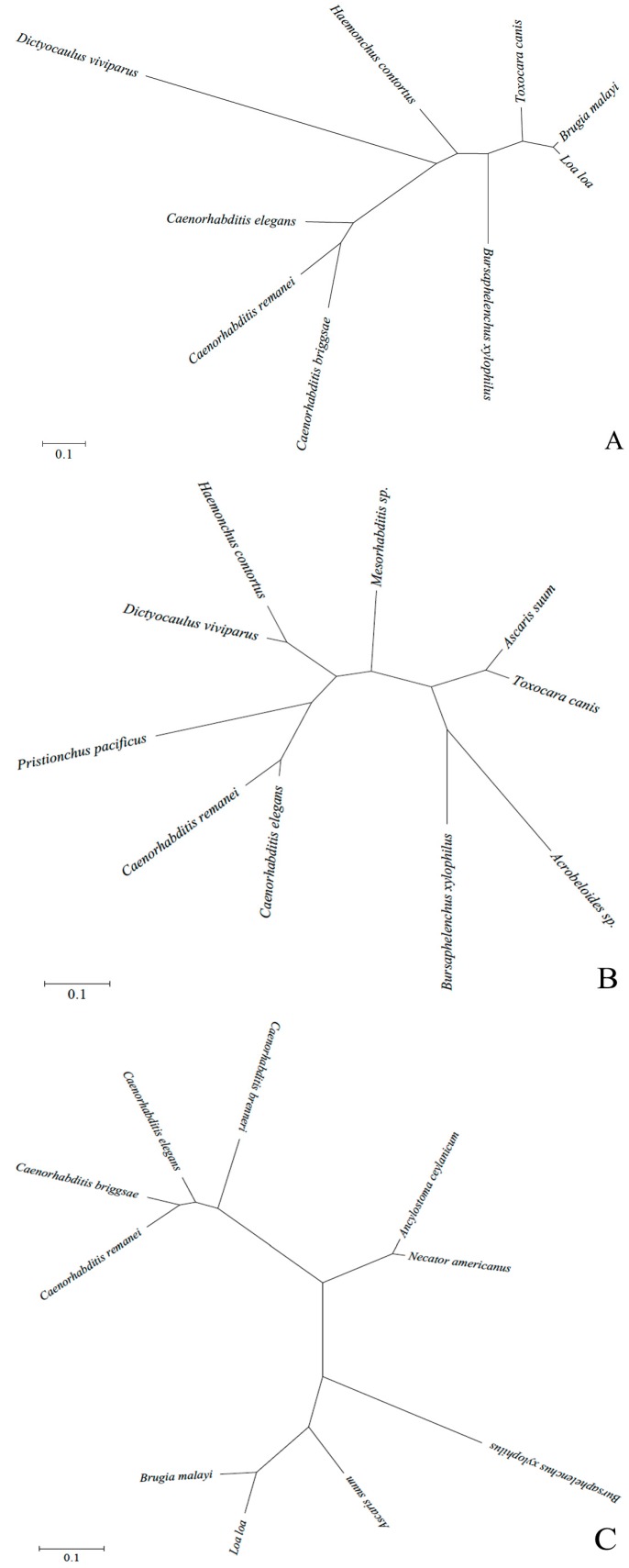
Analysis on phylogenetic trees of three deduced protein sequences with other organisms (same sequences as Figure 1). (**A**) Phylogenetic trees of the deduced Bx-DAF-11 protein sequences with other organisms’ DAF-11; (**B**) Phylogenetic trees of the deduced Bx-TAX-2 protein sequences with other organisms’ TAX-2; (**C**) Phylogenetic trees of the deduced Bx-TAX-4 protein sequences with other organisms’ TAX-4. One thousand bootstrap replicates were performed.

Sequence analysis showed that the coding sequence (CDS) of *Bx-daf-11* was 3216 bp encoding 1071 amino acids. CDS of *Bx-tax-2* was 2253 bp encoding 750 amino acids. CDS of *Bx-tax-4* was 1872 bp encoding 623 amino acids. Blastp results showed that the deduced amino acid sequence of Bx-DAF-11, Bx-TAX-2, and Bx-TAX-4 have a relatively high level of identity with the DAF-11, TAX-2, and TAX-4 protein of several nematodes. On this basis, selected homologous amino acid sequences were downloaded from NCBI. Multiple sequence alignments (Figure 1A–C) were performed. In addition, phylogenetic trees were constructed (Figure 2A–C).

### 2.2. Bioinformatics Analysis of Deduced Proteins

The protein molecular formula of Bx-DAF-11, Bx-TAX-2, and Bx-TAX-4 were C_5372_H_8369_N_1435_O_1631_S_64_, C_3877_H_6104_N_1058_O_1139_S_27_, and C_3219_H_5078_N_860_O_929_S_25_, respectively. Their deduced isoelectric points were 5.07, 8.72, and 6.76, respectively. The putative molecular masses were 121, 87, and 71 kDa, respectively. No signal peptide sequence was found by SignalP analysis. Results of TMHMM analysis showed that there were three transmembrane regions in both Bx-TAX-2 and Bx-TAX-4, one transmembrane region in Bx-DAF-11. This illustrated that Bx-DAF-11, Bx-TAX-2, and Bx-TAX-4 were membrane-binding proteins. Results of MotifFinder analysis showed that there was an adenylate and guanylate cyclase catalytic domain, an atrial natriuretic factor receptor domain, a protein tyrosine kinase domain, and an NO-binding-associated domain in Bx-DAF-11. There was a cyclic nucleotide-binding domain and an ion transport protein domain in Bx-TAX-2. There was also a C-terminal leucine zipper domain of cyclic nucleotide-gated channels, an ion transport protein, and a cyclic nucleotide-binding domain in Bx-TAX-4.

### 2.3. Analysis of Transcript Abundance at Low Temperature

**Figure 3:**
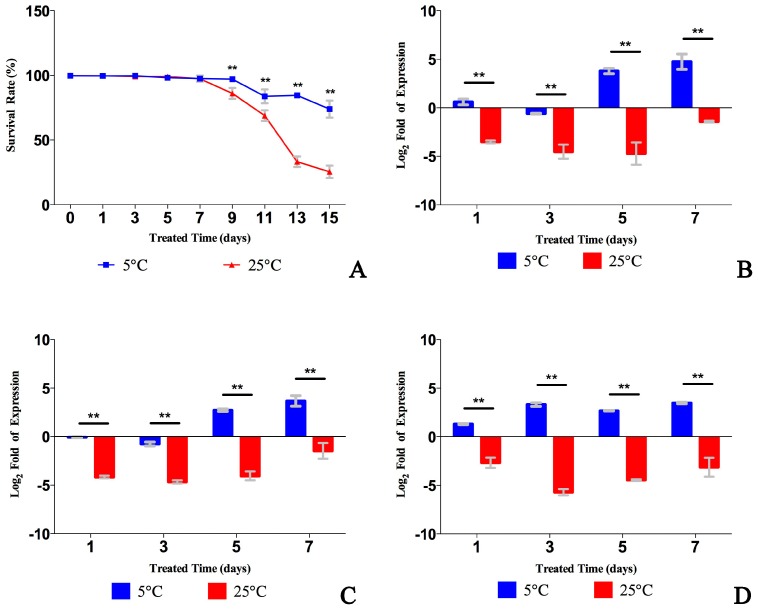
Survival rates of *B. xylophilus* and transcript abundance of three genes at 5 °C and 25 °C. (**A**) *B. xylophilus* showed higher survival rates at 5 °C than at 25 °C; (**B**) *Bx-daf-11* revealed higher transcript levels at 5 °C than 25 °C over 7 days; (**C**) *Bx-tax-2* revealed higher transcript levels at 5 °C than 25 °C over 7 days; (**D**) *Bx-tax-4* revealed higher transcript levels at 5 °C than 25 °C over 7 days. Data represent mean values ± standard deviation (SD) from different repetitions. Asterisks indicate statistically significant differences (** *p* < 0.001, Student’s *t*-test) were found between 5 °C and 25 °C.

The survival rates of *B. xylophilus* were calculated at 5 °C (low temperature) and 25 °C (regular temperature) every 2 days over 15 days. We found that the lifespan of *B. xylophilus* was extended at 5 °C (Figure 3A). To validate a relationship between *B. xylophilus* survival and the cGMP pathway transcript levels, we measured transcript levels of *Bx-daf-11*, *Bx-tax-2*, and *Bx-tax-4* at 5 °C and 25 °C for 1, 3, 5 and 7 days, respectively, with quantitative real-time PCR (qPCR). Surprisingly, three cGMP genes of *B. xylophilus* revealed different transcript patterns from *C. elegans* cGMP genes [17]. *Bx-daf-11*, *Bx-tax-2* and *Bx-tax-4* revealed higher transcript levels at low temperature than regular temperature over 7 days (Figure 3B–D).

### 2.4. RNAi of Bx-DAF-11, Bx-TAX-2, and Bx-TAX-4

**Figure 4:**
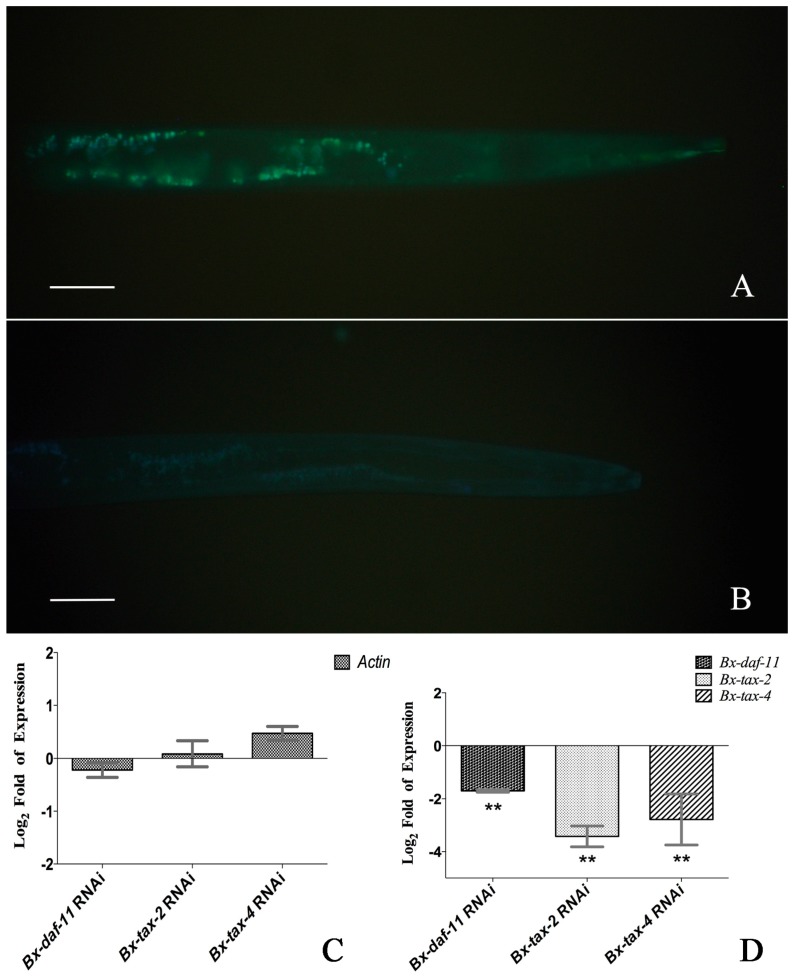
RNAi efficiency of three genes. (**A**) *B. xylophilus* soaked in fluorescein isothiocyanate (FITC) revealed green fluorescent signal; Scale bars = 20 μm; (**B**) *B. xylophilus* soaked in ddH_2_O (FITC-free) revealed no signal; Scale bars = 20 µm; (**C**) The *Bx-daf-11*, *Bx-tax-2*, and *Bx-tax-4* dsRNA had no obvious effect on the transcript level of *Actin*; (**D**) *Bx-daf-11*, *Bx-tax-2*, and *Bx-tax-4* transcript level log_2_ (RNAi-treated/RNAi-free) fold of *B. xylophilus*. Data represent mean values ± SD from different repetitions. Asterisks indicate statistically significant differences (** *p* < 0.001, Student’s *t*-test) were found between the dsRNA-treated group and dsRNA-free group.

The RNAi of *Bx-daf-11*, *Bx-tax-2*, and *Bx-tax-4* was performed for mixed-stage *B. xylophilus* as the method described by Feng Wang et al. (2012) [23] to allow nematodes to absorb double-stranded RNA (dsRNA). The patterns of fluorescein isothiocyanate (FITC) uptake observed here for the nematodes soaked in FITC solution were performed as described by Feng Wang et al. [23] (Figure 4A,B). qPCR analysis was utilized to examine the efficiency of *Bx-daf-11*, *Bx-tax-2*, and *Bx-tax-4* RNAi in *B. xylophilus*. The *Bx-daf-11*, *Bx-tax-2*, and *Bx-tax-4* dsRNA had no obvious effect on the transcript level of *Actin* (Figure 4C), while significant silencing was found after soaking in the relevant dsRNA (Figure 4D). These results indicated that the RNAi soaking method was potent and specific for *B. xylophilus*.

**Figure 5:**
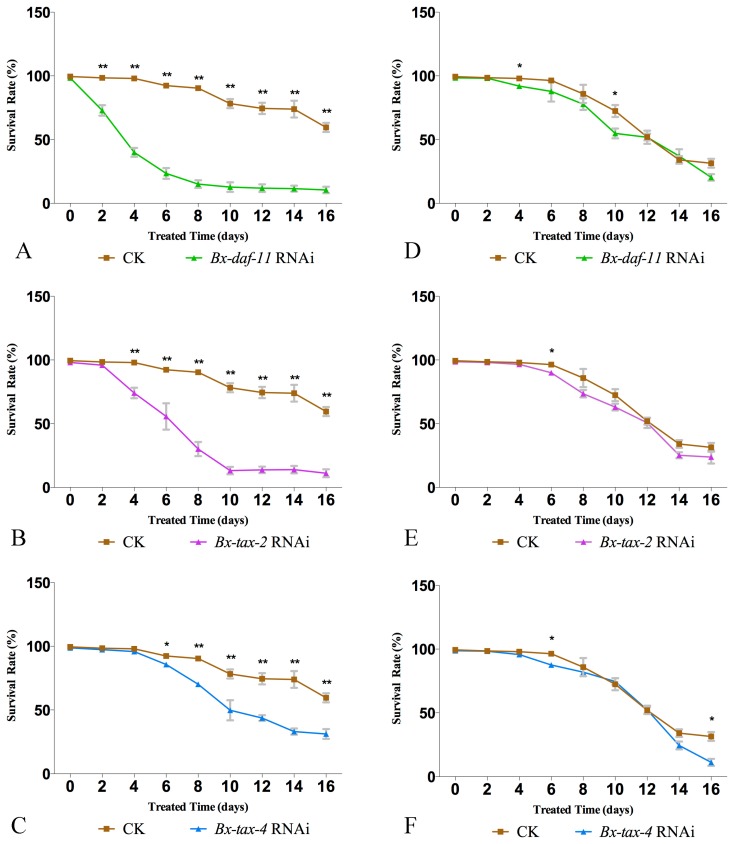
*B. xylophilus* revealed significantly different survival rates between the CK (dsRNA-free groups) and dsRNA-treated groups at 5 °C while few differences in survival rates between the CK and dsRNA-treated groups can be detected at 25 °C. (**A**) *Bx-daf-11* dsRNA-treated *B. xylophilus* showed a decreased survival rate at 5 °C; (**B**) *Bx-tax-2* dsRNA-treated *B. xylophilus* showed a decreased survival rate at 5 °C; (**C**) *Bx-tax-4* dsRNA-treated *B. xylophilus* showed a decreased survival rate at 5 °C; (**D**) CK and *Bx-daf-11* dsRNA-treated *B. xylophilus* showed few differences in survival rate at 25 °C; (**E**) CK and *Bx-tax-2* dsRNA-treated *B. xylophilus* showed few differences in survival rate at 25 °C; (**F**) CK and *Bx-tax-4* dsRNA-treated *B. xylophilus* showed few differences in survival rate at 25 °C. Data represent mean values ± SD from different repetitions. Asterisks indicate that statistically significant differences (* *p* < 0.01, ** *p* < 0.001, Student’s *t*-test) were found between the CK and dsRNA-treated groups.

The survival rates of dsRNA-treated group and the CK group (dsRNA-free) *B. xylophilus* were calculated every 2 days over 16 days in 5 °C and 25 °C, respectively. The result showed that the survival rates of *B. xylophilus* revealed a significant decrease after RNAi of *Bx-daf-11*, *Bx-tax-2*, and *Bx-tax-4* at 5 °C, while few differences in survival rates between the dsRNA-treated group and the dsRNA-free group could be detected at 25 °C (Figure 5). This indicated *Bx-daf-11*, *Bx-tax-2*, and *Bx-tax-4* play key roles in the process of low-temperature-induced lifespan extension in *B. xylophilus*. RNAi of the three genes can significantly shorten the lifespan of *B. xylophilus* at low temperature.

## 3. Discussion

It is commonly known that both homeotherms and poikilotherms can have extended lifespans at lower body temperatures. Cold-dependent lifespan extension is not a passive thermodynamic process but an active one that can be promoted by genetic programs at low temperatures [13]. In this study, we proved this theory in PWN through the fact that low-temperature-induced lifespan extension is also a genetic process regulated by the cGMP pathway in *B. xylophilus*. The past decade has witnessed a rapid progress in the understanding of how high temperature influences lifespan of *B. xylophilus* [23,24,25]. However, little is known about how low temperature promotes longevity.

In this article, the CDS of three cGMP pathway genes *Bx-daf-11*, *Bx-tax-2*, and *Bx-tax-4* were cloned and analyzed from *B. xylophilus*. Sequence analyzing indicated that the deduced amino acid sequences of Bx-DAF-11, Bx-TAX-2, and Bx-TAX-4 showed a high level of identity with other nematodes. Motif analysis revealed that *Bx-daf-11* may encode a membrane-bound form of guanylate cyclase, which has an ability to bind to extracellular ligands and synthesizing cGMP. *Bx-tax-2* and *Bx-tax-4* may encode a membrane-bound ion-gated channel. In addition, *Bx-daf-11*, *Bx-tax-2*, and *Bx-tax-4* revealed a higher transcript abundance under low temperature. Furthermore, the RNAi method was utilized to study the functions of *Bx-daf-11*, *Bx-tax-2*, and *Bx-tax-4* against low temperature. The results indicated that low temperature extended lifespan of *B. xylophilus* through three genes of the cGMP pathway. Considering all these factors, we hypothesized that low temperature upregulated the transcription of *Bx-daf-11* by binding with extracellular ligand. *Bx-daf-11* synthesized cGMP in the cytoplasm, which promoted the transcript level of *Bx-tax-2* and *Bx-tax-4*, encoding the membrane-bound ion-gated channel. Then, the ion concentration changed so that other physiological and biochemical activities were regulated to extend the lifespan of *B. xylophilus*.

Few articles about genetic mechanisms of low-temperature-induced lifespan extension in this devastating nematode have been published. In early autumn, low temperature as an environmental signal induces adult PWN to enter winter diapause in the host. Second-stage propagative juveniles (J_2_) turn into specialized third-stage dauer larva (DL_3_). DL_3_ accumulate around chambers of its vector beetles *Monochamus alternatus* [26]. Adult beetles emerge in the following spring. Developmentally arrested specialized fourth-stage dauer larvae (DL_4_) enter the tracheae of the beetle. These DL_4_ will be vectored to other pine trees by the beetle [27]. Extended lifespan extension is one of the characteristics of DL_3_ [12]. This study illustrates that three genes of the cGMP pathway are necessary for the low-temperature-induced lifespan extension in *B. xylophilus*, and these three genes are potential targets for the control of this destructive plant parasite nematode. This conclusion provides considerable guidance to the research about low-temperature-induced dauer formation of *B. xylophilus* in late summer and early autumn. However, future efforts are needed to discover other unknown genes and pathways related to low-temperature-induced lifespan extension in *B. xylophilus*.

## 4. Materials and Methods

### 4.1. Nematode Culture and Extraction

*B. xylophilus*, maintained in the Forestry Protection Laboratory of Northeast Forestry University, Harbin, China, were kindly provided by the Chinese Academy of Forestry, Beijing, China. The nematodes were cultured on *Botrytis cinerea* for 5–7 days at 25 °C. The Baermann funnel technique was used to isolate *B. xylophilus* from potato dextrose agar medium plates.

### 4.2. RNA Extraction and Cloning of Bx-daf-11, Bx-tax-2, and Bx-tax-4

The collected nematodes were ground into powder by a grinding rod after adding liquid nitrogen. Total RNA of nematodes was extracted with Trizol (Invitrogen, Carlsbad, CA, USA) as described by Wang, 2012 [23]. Then, RNA was treated with DNAse to remove possible DNA contamination (Promega, Madison, WI, USA). Double-stranded cDNA was obtained using GoTaq 2-Step RT-qPCR System (Promega, Madison, WI, USA) according to instructions of the manufacturer. The *daf-11*, *tax-2*, and *tax-4* sequences of *C. elegans* were used as the search sequence to BLAST the genomic data of *B. xylophilus* [22]. The homologous sequences of *daf-11*, *tax-2*, and *tax-4* were identified and named *Bx-daf-11*, *Bx-tax-2*, and *Bx-tax-4*, respectively. PCR primers covering CDS Bx-daf-11-F, Bx-daf-11-R, Bx-tax-2-F, Bx-tax-2-R, Bx-tax-4-F, and Bx-tax-4-R were designed for each sequence (Table 1). PCR products were sent to Sangon Biotech Company (Shanghai, China) for sequencing.

### 4.3. Cold Treatment and Survival Rate Counting

About 10,000 nematodes in 10 mL ddH_2_O were mixed evenly. A suspension (0.1 mL) containing about 80–120 nematodes were transferred to 1.5 mL centrifuge tubes with 1 mL of distilled water. The tubes were separated into two groups: one group was cultured at 5 °C and another at 25 °C. The survival rates of nematodes were calculated by assessing movement response to mechanical stimulation every 2 days over 15 days for each group by transferring nematodes to a microtiter plate, then observed through a dissecting microscope. Temperatures (5 °C and 25 °C) were maintained by two climatic cabinets (Bluepard, Shanghai, China).

### 4.4. Bioinformatic Analysis

The ORF Finder (available online: https://www.ncbi.nlm.nih.gov/orffinder/) was used to translate the complete protein CDS sequence into amino acids. Homologous *Bx-daf-11*, *Bx-tax-2*, and *Bx-tax-4* amino acid sequences of other organisms were obtained from National Center for Biotechnology Information with BLASTP (available online: https://blast.ncbi.nlm.nih.gov/Blast.cgi). Multiple sequence alignments of amino sequences were carried out using Geneious Basic 3.6.1 and phylogenetic analyses of Bx-DAF-11, Bx-TAX-2, and Bx-TAX-4 were performed with Mega 7.0 software using a maximum likelihood tree. Deduced protein molecular formula, molecular weights, and isoelectric point calculations were studied in Expasy (available online: http://web.expasy.org/protparam/). The GenomeNet Database (available online: http://www.genome.jp/tools/motif/) was used to search the motifs of Bx-DAF-11, Bx-TAX-2, and Bx-TAX-4. The bioinformatics website Center for Biological Sequence was used to analyze Predictions of Transmembrane helices (available online: http://www.cbs.dtu.dk/services/TMHMM/) and signal peptide (available online: http://www.cbs.dtu.dk/services/SignalP/).

### 4.5. Analysis of Transcript Abundance

About 90,000 nematodes were mixed evenly in 9 mL distilled water. Then, suspensions were separated into nine 1.5 mL centrifuge tubes equally. Four tubes were cultured at 5 °C for 1, 3, 5 and 7 days. The other four tubes were cultured at 25 °C for 1, 3, 5 and 7 days. We extracted the RNA of these eight tubes and synthesized it into cDNA using the method as described above after the incubation was finished. One tube was used to extract the RNA and synthesize that RNA into cDNA directly (no treated group). We used the cycle threshold data to calculate the fold of relative transcript abundance (each treated time point/no treated group). *Bx-daf-11*, *Bx-tax-2*, and *Bx-tax-4* gene-specific primers q-Bx-daf-11-F, q-Bx-daf-11-R, q-Bx-tax-2-F, q-Bx-tax-2-R, q-Bx-tax-4-F, and q-Bx-tax-4-R (Table 1) were utilized to amplify the product with the program: first step: 95 °C for 2 min, second step: 95 °C for 15 s and 60 °C for 1 min, in 40 cycles. The 28S gene was used as an internal control (Table 1). qPCR was analyzed with three replicates as three independent trials.

### 4.6. RNAi of Bx-daf-11, Bx-tax-2, and Bx-tax-4

In this research, RNAi method was utilized to study the functions of *Bx-daf-11*, *Bx-tax-2*, and *Bx-tax-4* as outlined in Feng Wang et al. [23]. Mixed-staged *B. xylophilus* (a mixture of adult and juvenile nematodes in a male-to-female-to-juvenile ratio of approximately 1:1:2) was used in this research. dsRNA was obtained using the MAXIscript T7/T3 RNA Synthesis Kit (Ambion, Tokyo, Japan) with the following primers: i-Bx-daf-11-F, i-Bx-daf-11-R, i-Bx-tax-2-F, i-Bx-tax-2-R, i-Bx-tax-4-F, and i-Bx-tax-4-R (Table 1). T7 promoter sequences in the RNAi primers are underlined (Table 1). The nematodes were soaked in ddH_2_O with 2 mg/mL dsRNA corresponding to the *Bx-daf-11*, *Bx-tax-2*, and *Bx-tax-4* sequence. The size of *Bx-daf-11*, *Bx-tax-2*, and *Bx-tax-4* dsRNA for RNAi is 306 bp, 378 bp, and 361 bp, respectively. The uptake of the dsRNA was monitored by a final nematode treatment of ddH_2_O containing 1 mg/mL FITC for 24 h. Pictures of nematodes were captured using a fluorescence microscope (Olympus, Tokyo, Japan). The CK nematodes were soaked in ddH_2_O only. After intermittent stirring for 24 h at 25 °C, the nematodes were washed with ddH_2_O to remove the external dsRNA and then observed fluoroscopically to detect the uptake of FITC.

The RNAi-treated nematodes were divided into two groups: The first group was used to examine the efficiency of RNAi with qPCR, and the second one was used to calculate survival rate under 5 and 25 °C environments. Temperature was provided by two climatic cabinets as mentioned above. Total RNA was extracted from the CK nematodes and the dsRNA-treated nematodes after intermittent stirring for 24 h. qPCR was then performed as mentioned above with q-Bx-daf-11-F, q-Bx-daf-11-R, q-Bx-tax-2-F, q-Bx-tax-2-R, q-Bx-tax-4-F, q-Bx-tax-4-R, q-Actin-F, and q-Actin-R. About 100 nematodes in 1 mL of ddH_2_O within each 1.5 mL centrifuge tube performed in triplicate were used to calculate the survival rates of nematodes every 2 days over 16 days.

### 4.7. Statistical Analysis

All assays were studied with three replicates as three independent trials. Microsoft Excel was utilized to calculate the mean ± SD of three independent experiments. SPSS Statistics 20.0.0 software (Shanghai, China) was applied to determine the statistical significance with the paired *t*-tests. Statistically significant differences are indicated with asterisks (* *p* < 0.01, ** *p* < 0.001, Student’s *t*-test).

## 5. Conclusions

A low temperature extends the lifespan of *B. xylophilus* by upregulating *Bx-daf-11*, *Bx-tax-2*, and *Bx-tax-4*. This is different from the expression pattern of *C. elegans* [20]. Bioinformatic analysis showed that deduced proteins of *Bx-daf-11*, *Bx-tax-2*, and *Bx-tax-4* have similar molecular characteristics with orthologous genes *daf-11*, *tax-2*, and *tax-4* [15,19,20,21]. RNAi of three genes of the cGMP pathway can significantly shorten the lifespan of *B. xylophilus* at low temperatures. The three genes are the potential targets for the control of this devastating plant parasite nematode.

## Figures and Tables

**Table 1:** Primers used in this study.

Name of Primer	Sequence (5′–3′)	Reference
Bx-daf-11-F	GATGCGATCCAGGTTTCTAC	This study
Bx-daf-11-R	TAATGTTACCGTTCCTCCGA	This study
Bx-tax-2-F	TCTCCCAATACTCCACAAGT	This study
Bx-tax-2-R	TTGGGTAACTGAGCAGAACT	This study
Bx-tax-4-F	TGTCACCAACATGAATGGAC	This study
Bx-tax-4-R	GGTGATGACTTCATCGTCTG	This study
q-Bx-daf-11-F	TGTTGGGACAATTGGTCAGG	This study
q-Bx-daf-11-R	TCACATTGTCATGGATTAACTGC	This study
q-Bx-tax-2-F	TGTGGACAATCAGTCGGAGA	This study
q-Bx-tax-2-F	CCAGGCCATGTAACTTTTGC	This study
q-Bx-tax-4-F	AACTCACACAGGGTTTCTGG	This study
q-Bx-tax-4-R	ACGTAGTCATGTAATCCAATGGAAG	This study
28S-F	TACGATCGGTGTTCGTTGC	Qiaoli Chen et al. [28]
28S-R	CTCACATCGTCGACATCCAA	Qiaoli Chen et al. [28]
i-Bx-daf-11-F	GCTAATACGACTCACTATAGGGATGCGCGTTCATGGGATTTTTG	This study
i-Bx-daf-11-R	AGTAATACGACTCACTATAGGGATCGCATGTCCCTTTTGTCGAAC	This study
i-Bx-tax-2-F	GCTAATACGACTCACTATAGGGATTCACTTAAGCGCGAGAGATG	This study
i-Bx-tax-2-R	AGTAATACGACTCACTATAGGGATCAAGGCGTTGTAGAGAAAGGC	This study
i-Bx-tax-4-F	GCTAATACGACTCACTATAGGGATGAGCAAGGGCTTCTGGTTAG	This study
i-Bx-tax-4-R	AGTAATACGACTCACTATAGGGATCCATCGGGGAGTGACTGTTTG	This study
q-Actin-F	GAAAGAGGGCCGGAAGAG	Jacob J et al. [29]
q-Actin-R	AGATCGTCCGCGACATAAAG	Jacob J et al. [29]

Note: T7 promoter sequences in the RNAi primers are underlined.

## Abbreviations

PWN	pine wood nematode
RNAi	RNA interference
cGMP	cyclic guanosine monophosphate
CDS	coding sequence
SD	standard deviation
qPCR	quantitative real-time PCR
dsRNA	double-stranded RNA
FITC	fluorescein isothiocyanate
J_2_	second-stage propagative juveniles
DL_3_	specialized third-stage dauer larva
DL_4_	specialized forth-stage dauer larva
